# MicroRNA-mediated regulation of target genes in several brain regions is correlated to both microRNA-targeting-specific promoter methylation and differential microRNA expression

**DOI:** 10.1186/1756-0381-6-11

**Published:** 2013-05-31

**Authors:** Y-h Taguchi

**Affiliations:** 1 Department of Physics, Chuo University, Tokyo 112-8551, Japan

**Keywords:** MicroRNA, Target gene regulation, Brain regions, Promoter methylation, Pathway analysis

## Abstract

**Background:**

Public domain databases nowadays provide multiple layers of genome-wide data e.g., promoter methylation, mRNA expression, and miRNA expression and should enable integrative modeling of the mechanisms of regulation of gene expression. However, researches along this line were not frequently executed.

**Results:**

Here, the public domain dataset of mRNA expression, microRNA (miRNA) expression and promoter methylation patterns in four regions, the frontal cortex, temporal cortex, pons and cerebellum, of human brain were sourced from the National Center for Biotechnology Informations gene expression omnibus, and reanalyzed computationally. A large number of miRNA-mediated regulation of target genes and miRNA-targeting-specific promoter methylation were identified in the six pairwise comparisons among the four brain regions. The miRNA-mediated regulation of target genes was found to be highly correlated with one or both of miRNA-targeting-specific promoter methylation and differential miRNA expression. Genes enriched for Kyoto Encyclopedia of Genes and Genomes (KEGG) pathways that were related to brain function and/or development were found among the target genes of miRNAs whose differential expression patterns were highly correlated with the miRNA-mediated regulation of their target genes.

**Conclusions:**

The combinatorial analysis of miRNA-mediated regulation of target genes, miRNA-targeting-specific promoter methylation and differential miRNA expression can help reveal the brain region-specific contributions of miRNAs to brain function and development.

## Background

miRNAs are short non-coding RNAs that are believed to suppress target gene expression through the binding of miRNA “seed” regions to complementary sequences of 3’ untranslated regions (UTR) of target genes [1]. miRNAs are generally assumed to regulate cellular processes related to animal development [2] and cellular differentiation, and have been implicated in several diseases, including cancer. Thus, miRNAs have been put forth as candidates for tumor suppression [3] and cancer biomarkers [4]. miRNAs are also known to be involved in reprogramming [5]. As such, miRNAs are considered to play critical roles in a wide range of biological processes.

Recently, miRNA expression in the brain has attracted the interest of many researchers [6-9]. Although there are extensive researches about miRNA regulation of target genes [6,7], it is generally believed that the expression of many genes is regulated by miRNAs indirectly [10]. In this sense, in order to understand miRNA regulation of gene expression in brain regions, it is also important to understand the mechanisms by which such regulation occurs.

Together with miRNAs, transcription factors (TFs) bind to promoter regions and cooperatively regulate miRNA target genes [11-15]. TFs form a protein complex that binds to gene promoters during the initiation of transcription. Since there are many TFs known to regulate biological processes in regions of the brain [16-18], it is natural to investigate the combinatorial effects of TFs and miRNA gene regulation in the brain [19,20]. In contrast to what is known about cooperative regulation by miRNA and TFs, investigations of gene coregulation mediated by both miRNA and promoter methylation are limited; however, siRNA-induced promoter methylation in CpG islands has been reported [21-24]. Promoter methylation is generally thought to suppress gene expression [25]. Suppression of gene expression by promoter methylation is often important. For example, aberrant promoter methylation is often related to cancers [26,27]. Promoter methylation also plays critical roles in reprogramming [28].

Despite the known importance of promoter methylation, the relationship between promoter methylation and miRNA-mediated gene regulation has received little attention. However, it was recently shown that promoters of genes not targeted by miRNAs have higher levels of methylation [29]. We recently found that miRNA-targeting-specific promoter methylation takes place in many cell lines [30,31]. miRNA-targeting-specific promoter methylation refers to the association between 3 BUTR miRNA targetting and promoter methylation levels for a given gene.

In this paper, we report that miRNA-targeting-specific promoter methylaion also exists between distinct brain-regions in a brain-region specific manner. Considered brain regions are frontal cortex, temporal cortex, pons, and cerebellum [32]. The frontal cortex is located at the front of the head in human. It is considered to be the hub of most higher functions and understanding, and is believed to govern most behavioral traits, motor skills, and problem solving tactics [33]. The temporal cortex is located in the lower right and left regions of the brain, and is involved in hearing, understanding languages, face recognition, and certain memory functions [34]. The cerebellum is located in the lower region at the back of the brain, and is central to motion control [35]. Finally, the pons is located in the center of these three regions and mediates information transfer between several other brain regions, including the cortex and cerebellum [32]. Given the diverse functions of these brain regions, I hypothesized that miRNA-targeting-specific promoter methylation would occur in a region-specific manner. Not only did I determine that patterns of miRNA regulation were indeed brain-region specific, I also revealed that some miRNA regulation of target genes turned out to be controlled by not only differential miRNA expression itself but also miRNA-targeting-specific promoter methylaion. In addition, target genes of miRNAs whose regulation was significantly correlated to differential miRNA expression were also found to be enriched for brain-region-specific functions and related KEGG pathways.

## Methods

### Patterns of miRNA and mRNA expression and promoter methylation

Datasets used in this study were downloaded from Gene Expression Omnibus (GEO) under GEO ID GSE15745. These included miRNA and mRNA expression, and promoter methylation data from four distinct brain regions (frontal cortex, temporal cortex, pons and cerebellum) in 150 human subjects [36], which had been analyzed in detail in connection with genomic variants, such as single nucleotide polymorphisms and copy number variants; however, miRNA expression had not been analyzed previously [36]. Thus, in total, 600 tissue samples were included. Processed signals were used without any further normalization. For more details about data processing and analysis, see the Supplementary Document (see Additional file 1).

## Results and discussion

In this section, I will discuss the mutual relationships between miRNA-related features and their biological meaning.

### Mutual relationships between miRNA-mediated regulation of genes, miRNA-targeting-specific promoter methylation, and differential miRNA expression

**Figure 1:**
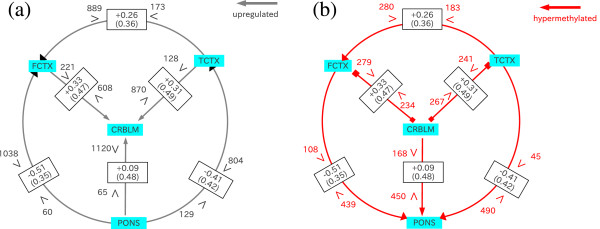
**Schematic illustration of the relationship between miRNA-mediated gene regulation and miRNA-targeted-specific promoter methylation.** Arrows/segments indicate up/downregulation of miRNA target genes (**a**) and miRNA-targeted-specific promoter methylation (**b**). Black (red) numbers next to inequality signs are the averaged number of miRNAs whose target genes are significantly up/downregulated (whose target genes promoters are hyper/hypomethylated). TCTX refers to the temporal cortex, FCTX to the frontal cortes, CRBLM to the cerebellum, and PONS to the pons. For example, there are 280 (183) miRNAs whose target gene promoters are significantly hyper(hypo)methylated in FCFX compared to TCTX. Similarly, there are 889 (173) miRNAs whose target genes are significantly up(down)regulated in FCFX compared to TCTX. Since 280 is larger than 183, gene promoters in FCFX are considered to be more hypermethylated than those in TCTX based on the miRNA-centric-view, thus the red arrow directs the reader from TCTX to FCTX. Likewise, because 889 is larger than 173, genes in FCFX are considered to be upregulated when compared to TCTX, thus the black arrow directs the reader from TCTX to FCTX. The numbers in the rectangle indicate Spearman correlation coefficients between miRNA-mediated gene regulation and miRNA-targeted-specific promoter methylation, $\rho_{\ell \ell^{\prime }}^{\text{mRNA},\text{Methyl.}}$. Standard deviations of Spearman correlation coefficients, $\Delta \rho_{\ell \ell^{\prime }}^{\text{mRNA},\text{Methyl.}}$ are shown in parentheses.

I investigated miRNA-mediated gene regulation and miRNA-targeting-specific promoter methylation in the frontal cortex, temporal cortex, pons, and cerebellum of the human brain, based on the *P*-values, $P_{\text{mj},<}^{\ell \ell }$s or $P_{\text{mj},>}^{\ell \ell^{\prime }}$s, which were used to estimate miRNA-mediated gene regulation and miRNA-targeting-specific promoter methylation. Figure 1 illustrates the results of this analysis. It is clear that target genes of a substantial number of miRNAs are up/downregulated between these four brain regions. It is also evident that some miRNA target genes are differentially methylated between these four brain regions. This strongly suggests that both miRNA-mediated gene regulation and miRNA-targeting-specific promoter methylation play critical roles in the development and function of these four brain regions. For example, from the miRNA-centric point of view (Figure 1), compared to the other three brain regions investigated, the pons has more genes with hypermethylated promoters and lower expression levels, although these characteristics are not always associated. This observation is consistent with the general belief that the hypermethylation of promoters is associated with reduced expression. This also signifies that mRNA expression in the pons is distinct from the other three brain regions.

### Mutual relationships between miRNA-mediated regulation of target genes and miRNA-targeting-specific promoter methylation

In order to understand the mutual relationship between miRNA-mediated gene regulation and miRNA-targeting-specific promoter methylation, I computed the correlation coefficient of the mean rank of *P*-values, $\rho_{\ell \ell^{\prime }}^{\text{mRNA},\text{Methyl.}}$, for six pairwise comparisons between the frontal cortex, temporal cortex, pons, and cerebellum (see Figure 1). Here, the means were taken over all samples in each brain region. Excluding a single pairwise comparison between the cerebellum and pons, correlation coefficients for the remaining five comparisons varied between 0.25 and 0.51. These values were considered to be sufficiently large taking into account the fact that the number of *P*-values in a given brain region is as large as *M*, the number of miRNAs comsidered. The *P*-values of each correlation coefficient are less than 2.2×10^−16^. This means, the correlation between miRNA-mediated gene regulation and miRNA-targeting-specific promoter methylation is highly significant independent of pairs of brain regions. The smallest correlation coefficients were observed in the cerebellum and pons. Although the correlation coefficient was large in aggregate (0.09), individual *P*-value was as small as 4×10^−5^, which is highly significant.

In order to confirm the correlation between miRNA-mediated gene regulation and miRNA-targeting-specific promoter methylation, the root mean squared averages of the correlation coefficients in each sample, $\Delta \rho_{\ell \ell^{\prime }}^{\text{mRNA},\text{Methyl.}}$, were also computed. Excluding pairwise comparisons for the frontal cortex and pons for which the absolute value of $\rho_{\ell \ell^{\prime }}^{\text{mRNA},\text{Methyl.}}$ was the maximum, $\Delta \rho_{\ell \ell^{\prime }}^{\text{mRNA},\text{Methyl.}}$ was larger than the absolute value of $\rho_{\ell \ell^{\prime }}^{\text{mRNA},\text{Methyl.}}$. This signifies that the correlation coefficients within each sample were not small, but that when averaged over all samples, the value was seemingly small because of the occurrence of both positive and negative correlations with equal probabilities. Thus, I conclude that miRNA-mediated regulation and miRNA-targeting-specific promoter methylation are significantly correlated. Worth noting is that the signs of correlation coefficients, $\rho_{\ell \ell^{\prime }}^{\text{mRNA},\text{Methyl.}}$, are neither definitively positive nor negative. One may think that they should be positive, as both promoter methylation and miRNA targeting should suppress gene expression. However, because genes targeted by miRNAs are expected to be downregulated (upregulated) only when miRNA itself is upregulated (downregulated), there is no reason to expect that the correlation coefficients between miRNA-mediated gene regulation and miRNA-targeting-specific promoter methylation should always take positive or negative values.

### Relationships between miRNA-mediated regulation of target genes, miRNA-targeting-specific promoter methylation and differential miRNA expression

In order to determine the relationship between miRNA-mediated gene regulation, $P_{\text{mj},<}^{\ell \ell^{\prime }}$ or $P_{\text{mj},>}^{\ell \ell^{\prime }}$, and differential expression of miRNA, $\log\left(\frac{x_{\text{mjℓ}}}{x_{\text{mj}\ell^{\prime }}}\right)$, the correlation coefficients were computed. However, these correlation coefficients were too small to be significant (not shown here). This seemingly contradicts the observed correlation between miRNA-mediated gene regulation and miRNA-targeted-specific promoter methylation.

Thus, in order to resolve this apparent discrepancy, I employed multivariate regression models between miRNA-mediated gene regulation, miRNA-targeting-specific promoter methylation, and differential miRNA expression, also considering both sample gender and age (see Methods). In contrast to the above discrepancy, depending upon the miRNA considered, I identified significant correlations between only selected variables that were included in the regression model. In other words, I found that all of the variables were not always correlated, but were instead selectively correlated. In order to quantize these correlations, for each miRNA, I picked out the combinations of variables that were significantly correlated (see Methods). Table 1 lists the miRNAs selected for each pair of brain regions based on the criterion described in the subsection, “The selection of miRNAs that significantly regulate target genes based on multiple regression” in Supplementary Document (see Additional file 1), i.e., miRNAs whose differential expression is significantly correlated to miRNA-mediated gene regulation. To our knowledge, this is the first time that miRNA gene regulation has been shown to be mediated by both differential miRNA expression and miRNA-targeting-specific promoter methylation.

**Table 1:** miRNAs that significantly regulate target genes

**CRBLM vs FCTX**		**CRBLM vs PONS**		**CRBLM vs TCTX**	
**Reciprocal**	**Nonreciprocal**	**Reciprocal**	**Nonreciprocal**	**Reciprocal**	**Nonreciprocal**
**hsa-miR-181c-5p**	**hsa-miR-200a-5p**	hsa-miR-20a-5p	**hsa-let-7b-5p**	**hsa-miR-210**	**hsa-miR-99a-5p**
hsa-miR-135a-5p	**hsa-miR-381**	hsa-miR-23a-3p	**hsa-let-7e-5p**		**hsa-miR-191-5p**
**hsa-miR-137** *	hsa-miR-202-3p *	**hsa-miR-148a-3p**	**hsa-miR-197-3p**		**hsa-miR-99b-5p** *
**hsa-miR-363-3p**	hsa-miR-561-3p	hsa-miR-10a-5p	**hsa-miR-181b-5p**		**hsa-miR-617**
hsa-miR-369-3p	hsa-miR-568	**hsa-miR-221–3p**	**hsa-let-7i-5p**		
hsa-miR-487a *	hsa-miR-618	hsa-miR-223–3p	**hsa-miR-9-5p**	FCFX vs PONS	
hsa-miR-514a-3p	**hsa-miR-630** *	**hsa-miR-1**	hsa-miR-126-3p	hsa-miR-365a-3p	**hsa-miR-302d-3p**
hsa-miR-553		**hsa-miR-133a**	hsa-miR-134	hsa-miR-378a-5p	**hsa-miR-432-5p**
hsa-miR-554		**hsa-miR-137** *	hsa-miR-154-3p		hsa-miR-595
hsa-miR-655		**hsa-miR-146a-5p**	**hsa-miR-299-5p**		
hsa-miR-421		**hsa-miR-452-5p**	**hsa-miR-99b-5p** *	FCTX vs TCTX	
		**hsa-miR-484**	hsa-miR-377-3p	has-miR-373-3p	**hsa-miR-24-3p**
		**hsa-miR-511**	hsa-miR-383		**hsa-miR-485-5p**
		**hsa-miR-515-5p**	hsa-miR-431-5p		**hsa-miR-766-3p**
		hsa-miR-571	hsa-miR-329		
		hsa-miR-549	**hsa-miR-485-3p**	PONS VS TCTX	
			hsa-miR-487a *	**hsa-miR-9-3p**	**hsa-miR-222-3p**
			hsa-miR-202-3p *	**hsa-miR-302a-3p**	**hsa-miR-125b-5p**
			**hsa-miR-432-3p**	hsa-miR-410	**hsa-miR-328**
			hsa-miR-495	hsa-miR-487b	hsa-miR-581
			hsa-miR-504	**hsa-miR-630** *	**hsa-miR-661**
			hsa-miR-505-3p		
			hsa-miR-563		
			hsa-miR-578		
			**hsa-miR-630** *		
			**hsa-miR-668**		

miRNAs predicted to regulate target genes based on six pairwise comparisons among four brain regions: the frontal cortex (FCTX), temporal cortex (TCTX), pons (PONS), and cerebellum (CRBLM). The labels “Reciprocal” and “nonreciprocal” indicate whether the observed relationship between miRNA expression and target gene mRNA was either reciprocal or nonreciprocal. Asterisked miRNAs appear more than once. Bold faced miRNAs were previously reported to be related to brain development/diseases [37-39]. See subsection “The selection of miRNAs that significantly regulate target genes based on multiple regression model” in Supplementary Document (see Additional file 1) for the detailed criterion of miRNAs selection.

### Biological meanings of findings

As can be seen in Table 1, miRNAs selected for each pair of brain regions are not unique, but rather divergent. Some of the listed miRNAs were previously reported to be important in specific brain regions. For example, Yao *et al* recently investigated miRNA expression in the rat cerebral cortex during brain development [37]. Many of the top 20 most highly expressed miRNAs identified by Yao *et al* at each of eight different developmental stages, ranging from early developmental stages to late post natal stages, were also significant in our dataset (rno-let-7b, 7e, 7i, rno-miR-181b, 99a/b, 9, 125b-5p, and 191). Yao *et al* also emphasized the importance of miR-137, the ortholog of the human miRNA, hsa-miR-137; this miRNA was found to be significant twice in our analysis, compared to the most of other miRNAs which were only identified as significant once. In addition, many of the miRNAs listed in Table 1 have also been previously implicated in brain diseases, including Alzheimers disease (AD), Parkinsons disease (PD), Huntingtons disease (HD), and various other neurodegenerative disorders [38,39]. This overlap lends support to the utility of our method for identifying miRNAs with potential functional relevance in the brain. Although there have been other investigations of brain miRNA expression, to our knowledge, I am the first to interrogate miRNA expression data across multiple brain regions.

In order to better understand the biological functions of the miRNA targets identified in our analysis, I employed pathway analysis (Table 2), which has been shown previously to be effective for miRNA target genes (e.g., [40,41]). For this purpose, I used DIANA-mirPath [42], which is a web tool developed for KEGG pathway enrichment analysis of miRNA target genes.

**Table 2:** miRNA target genes KEGG pathway enrichment

		**CRBLM**		**CRBLM**		**CRBLM**		**FCTX**		**FCTX**		**PONS**	
		**vs**		**vs**		**vs**		**vs**		**vs**		**vs**	
		**FCTX**		**PONS**		**TCTX**		**PONS**		**TCTX**		**TCTX**	
	**KEGG pathways**	**R**	**N**	**R**	**N**	**R**	**N**	**R**	**N**	**R**	**N**	**R**	**N**
1	**TGF- *β * signaling pathway**	○	○	○	○				○			○	
2	Glioma ∗	○	○	○	○	○			○				
3	**MAPK signaling pathway**	○	○	○	○						○		
4	Axon guidance ∗	○	○	○	○								
5	Phosphatidylinositol signaling system	○	○		○								
6	**mTOR signaling pathway**	○	○	○									
7	Adipocytokine signaling pathway	○	○		○		○						
8	Pancreatic cancer	○	○		○				○				
9	**Endocytosis**	○	○	○	○							○	
10	**Focal adhesion**	○	○	○	○							○	
11	Insulin signaling pathway	○	○	○	○								
12	Neurotrophin signaling pathway ∗	○	○	○	○								
13	Colorectal cancer	○	○	○	○								
14	Arrhythmogenic right ventricular cardiomyopathy (ARVC)	○	○	○	○						○		
15	**Wnt signaling pathway**	○	○	○	○		○				○		
16	Non-small cell lung cancer	○	○										
17	**Adherens junction**	○	○	○	○							○	
18	**ErbB signaling pathway**	○	○	○	○								
19	Pathways in cancer	○	○	○	○				○			○	
20	Glycosaminoglycan biosynthesis - heparan sulfate	○	○	○	○				○	○			
21	Type II diabetes mellitus	○	○										
22	Melanoma	○	○		○				○				
23	Renal cell carcinoma	○	○	○	○								
24	Inositol phosphate metabolism	○	○										
25	Chronic myeloid leukemia	○	○	○	○				○				
26	T cell receptor signaling pathway	○	○										
27	Small cell lung cancer	○	○	○									
28	Fc gamma R-mediated phagocytosis	○	○		○								
29	Prostate cancer	○	○	○									
30	Salivary secretion	○	○										
31	Osteoclast differentiation	○	○										
32	**Regulation of actin cytoskeleton**	○	○	○	○								
33	Endocrine and other factor-regulated calcium reabsorption	○	○										
34	**Lysine degradation**			○	○		○						
35	Circadian rhythm - mammal			○	○				○		○		
36	Glycosaminoglycan biosynthesis - chondroitin sulfate			○	○					○			
37	**N-Glycan biosynthesis**			○	○								
38	**Long-term depression**				○				○				
39	**Prion diseases**				○							○	
40	**ECM-receptor interaction**			○						○			
41	**Tight junction**				○						○		
42	Hypertrophic cardiomyopathy (HCM)				○						○		
43	Cell adhesion molecules (CAMs)						○	○					
44	Dilated cardiomyopathy				○						○		
45	Fatty acid biosynthesis	○											
46	**Long-term potentation**			○									
47	Ubiquitin mediated proteolysis			○									
48	Thyroid cancer			○									
49	Notch signaling pathway			○									
50	Mismatch repair			○									
51	Acute myeloid leukemia			○									
52	Glycosphingolipid biosynthesis - lacto and neolacto series			○									
53	Glycosaminoglycan biosynthesis - keratan sulfate				○								
54	Biotin metabolism				○								
55	Gap junction				○								
56	Gastric acid secretion				○								
57	Taurine and hypotaurine metabolism				○								
58	Aldosterone-regulated sodium reabsorption				○								
59	GnRH signaling pathway				○								
60	Metabolism of xenobiotics by cytochrome P450						○						
61	Mucin type O-Glycan biosynthesis								○				
62	Biosynthesis of unsaturated fatty acids									○			
63	Viral myocarditis										○		
64	Cytokine-cytokine receptor interaction											○	
65	Hematopoietic cell lineage											○	
66	Valine, leucine and isoleucine biosynthesis												○

KEGG pathways marked with ○ are enriched by target genes of miRNAs selected in Table 1. “R” and “N” indicate whether the relationship between miRNA expression and target gene mRNA is reciprocal (nonreciprocal). Pathways asterisked and bold faced are directly related to brain and neurons, respectively, and discussed in detail in the Supplementary Document (see Additional file 1).

Compared to the variation observed in the miRNAs listed in Table 1, KEGG pathways for miRNA targets (Table 2) are highly universal and biologically meaningful as shiwn in the followings. For example, Paul *et al*[43] measured and analyzed transcritpomes in the mouse cerebellum. Cells were classified into Purkinje cells (PCs) at postnatal days 3, 7, 14, 21, 28, 35, and 56 (P3, P7, P14, P21, P28, P35, and P56), and the mixture of Stellate/Basket cells (StCs/BKCs) at P14, P21, P28, P35, and P56. They conducted pathway enrichment analysis using KEGG pathways based on developmental gene expression of PCs and S/BCs. From this, they found that many pathways were enriched at several different time points. In their data, upregulated genes identified between P3-PCs and P7-PCs were enriched for pathways such as “axon guidance”, “regulation of actin cytoskeleton”, “gap junction”, and “tight junctions”, implicating roles for these genes in the early stages of circuit integration by PCs. These changes are accompanied by an upregulation of other pathways such as insulin, TGF- *β*, Hedgehog, and Wnt signaling, which are important for axon guidance. The upregulation of GnRH signaling, which is known to have a modulatory effect on cerebellar neurons and P53 signaling, and is important for PC survival was also observed during this time.

In P14-PCs, Paul *et al* also reported that pathways related to “long-term potentiation”, “long-term depression”, “JAK/STAT”, “VEGF”, and “mTOR signaling” were elevated, which correlate to the development of parallel fiber synapses. Between P28 and P56, the upregulation of pathways related to “CAMs”, “chondroitin sulfate biosynthesis”, “focal adhesion”, “cytokine receptor interaction”, and “extracellular matrix receptor interaction” (ECM-interaction) correlate with the maturation and stabilization of PC connectivity. In S/BCs a number of similar pathways are also activated. “axon guidance”, “tight junction”, “adherens junction”, “insulin signaling”, “ErbB”, and “spliceosome” pathways were upregulated in P14S/BCs, reflecting delayed axogenesis of BskC and StC after they enter the ML during the second postnatal week. However, between P28 and P35, similar to PC cells, pathways of “ECM-receptor interaction”, “CAMs”, “cytokine receptor interaction”, “neuroactive ligand receptor interactions” and “regulation of cytoskeleton” were activated. These listed pathways largely overlap those listed in Table 2. Although Paul *et al* mainly investigated the cerebellum, I studied the cerebellum, as well as the pons, and frontal and temporal cortex; thus, I investigated previous studies related to individual pathways listed in Table 2 one by one.

#### Pathways directly related to brain/nervous system

Some pathways listed in Table 2 are obviously related to the brain and/or nervous system. For example, “axon guidance” is definitely included in brain development. “Glioma” is a brain tumor and the “neurotrophin signaling pathway” is related to neural systems. Enrichments in these three pathways further supports the notion that the genes I have identified are indeed relevant to brain function and development. For additional discussion of other selected pathways, see the Supplementary Document (see Additional file 1).

### Other notable observations

For further discussion of the divergence of miRNAs selected vs. the uniformity of pathways selected, positive vs. negative correlations between miRNA expression and target gene expression, and possible biological explanations underlying coregulation by both miRNA and promoter methylation, see the Supplementary Document (see Additional file 1).

## Conclusion

In this paper, I demonstrated possible miRNA coreguation of target genes in brain regions by analyzing both differential miRNA expression and miRNA-targeting-specific promoter methylation. Selected miRNAs were enriched in brain-related KEGG pathways. Because this was simply descriptive and no mechanisms responsible for the cooperative regulation described above were presented, experiment-based follow up studies will be necessary to validate our findings.

## Competing interests

The author declares that they have no competing interests.

## Supplementary Material

Additional file 1 Supplementary Document: Supplementary information not included in the paper. Click here for file
