# Supplementary material for: MicroRNA-mediated regulation of target genes in several brain regions is correlated to both microRNA-targeting-specific promoter methylation and differential microRNA expression
Source: BioData Min. 2013 May 31;6:11. doi: 10.1186/1756-0381-6-11 (PMC3693885; doi:10.1186/1756-0381-6-11)
Supplement: Additional file 1 — Supplementary Document: Supplementary information not included in the paper. [file 1756-0381-6-11-S1.pdf]

Supplementary Document

Y-h. Taguchi*[1]

[1]Department of Physics, Chuo University, Tokyo 112-8551, Japan

Email: Y-h. Taguchi*- tag@granular.com;

*Corresponding author

Contents

1 Supplementary Methods **2**

1.1 Computation of the P-values for the miRNA-mediated regulation of target genes and miRNA-targeting-specific promoter methylation . . . 2

1.2 Comparison of the P-values for the distinct brain regions . . . 3

1.3 Estimation of the number of miRNAs with target genes that were significantly up/downregulated or the number of target gene promoters that were significantly hypo/hyper methylated between any two brain regions . . . 4

1.4 Rank correlation coefficients between P-values attributed to miRNA-mediated regulation of target genes and P-values attributed to miRNA-targeting-specific promoter methylation . . . 4

1.5 Multiple regression model between miRNA-mediated regulation of target genes, miRNA-targeting-specific promoter methylation, and differential miRNA expression, with the additional consideration of age and gender . . . 5

1.6 Selection of miRNAs that significantly regulated target genes based on multiple regression models . . . 6

1.7 KEGG pathway enrichment analysis of the selected miRNAs . . . 6

2 Supplementary Discussion **6**

2.1 Supplementary Discussion on Selected KEGG pathways . . . 6

2.1.1 TGF-β signaling pathway . . . 6

2.1.2 Long-term potentiation and depression . . . 7

2.1.3 The mitogen-activated protein kinase (MAPK) signaling pathway 7
2.1.4 mTOR signaling pathway 8
2.1.5 Wnt signaling pathway 8
2.1.6 ErbB signaling pathway 9
2.1.7 Lysine degradation 9
2.1.8 N-glycan biosynthesis 10
2.1.9 Tight junction and adherens junction 10
2.1.10 Regulation of the actin cytoskeleton 11
2.1.11 Prion disease 11
2.1.12 ECM-receptor interaction 11
2.1.13 Focal adhesion 11
2.1.14 Endocytosis 12
2.1.15 Other pathways 12
2.2 Divergence of the selected miRNAs and uniformity of the selected pathways 12
2.3 Positive and negative correlations between miRNA expression and target gene expression . . 13
2.4 Possible biological reasons for cooperative regulation of target genes by miRNAs and promoter methylation 13
2.5 Table 3 - Genes differently expressed between frontal cortex and cerebellum in TFG-β pathway 19

1 Supplementary Methods

1.1 Computation of the P-values for the miRNA-mediated regulation of target genes and miRNA-targeting-specific promoter methylation

We have previously described the use of the MiRaGE method to calculate P-values for the miRNA-mediated regulation of target genes for each miRNA [1, 2] and to attribute P-values for miRNA-targeting-specific promoter methylation [3,4]. Therefore, the method is explained only briefly below. First, a matrix $x_{ij\ell}$ that represents either the expression or promoter methylation of the ith gene at the ℓth region of the jth sample is prepared. Then, the differential expression/methylation is computed as follows,

$$\Delta x_{ij}^{\ell\ell'} \equiv \log\left(\frac{x_{ij\ell'}}{x_{ij\ell}}\right).$$

When $x_{ij\ell}$ is not positive definite, the cases that do not satisfy the positive definite condition can either be ignored or, alternatively,

$$\Delta x_{ij}^{\ell\ell'} \equiv x_{ij\ell'} - x_{ij\ell}$$

can be used. Next, define a set of target genes G_m of the mth miRNA and a set of genes G'_m not targeted by the mth miRNA but targeted by any other miRNA, based on simple seed match

$$G'_m \equiv \bigcup_{m' \neq m} G_{m'}$$

Then P-values to deny the null hypothesis are computed as

$$\left\{\Delta x_{ij}^{\ell\ell'} \mid i \in G_m\right\} = \left\{\Delta x_{ij}^{\ell\ell'} \mid i \in G'_m\right\}$$

in favor of

$$\left\{\Delta x_{ij}^{\ell\ell'} \mid i \in G_m\right\} < \left\{\Delta x_{ij}^{\ell\ell'} \mid i \in G'_m\right\}$$

or

$$\left\{\Delta x_{ij}^{\ell\ell'} \mid i \in G_m\right\} > \left\{\Delta x_{ij}^{\ell\ell'} \mid i \in G'_m\right\}$$

using any statistical tests. The P-values computed based on the former (latter) are denoted as $P_{mj,<}^{\ell\ell'}$ ($P_{mj,>}^{\ell\ell'}$). A smaller $P_{mj,<}^{\ell\ell'}$ ($P_{mj,>}^{\ell\ell'}$) indicates that the targets gene are more up(down)regulated or hyper(hypo)methylated in the ℓth tissue compared with in the ℓ'th tissue. In our implementation, we used the t test, the Wilcoxon rank sum test, and the Kolmogorov-Smirnov test to compute P-values. For more details, see my recent review [4] or the vignette package in the Bioconductor software [5]. Also one should notice that target genes of miRNAs whose target genes are significantly up/downregulated are not always up/downregulated. The statement that target genes of a miRNA are significantly up/downregulated simply means that majority of miRNA target genes are up/downregulated. Thus minority of target genes of miRNAs whose target genes are significantly up(down)regulated can always be down(up)regulated. This statement can be applied only to the set of whole target genes of a miRNA, not to individual gene in the set.

1.2 Comparison of the P-values for the distinct brain regions

For the pairwise comparisons among the frontal cortex, temporal cortex, pons, and cerebellum, P-values of both miRNA-mediated target regulation and miRNA-targeting-specific promoter methylation were attributed to each miRNA. For each sample, twelve P-values, i.e., for the six pairwise comparisons among the four brain regions of both miRNA-mediated target regulation and miRNA-targeting-specific promoter methylation, were attributed to each miRNA. Only the miRNAs that were among the human miRNAs in miRBase (release 18) were considered. The Wilcoxon rank-sum test was employed to compute the P-values. The number of miRNAs M was 1921.

1.3 Estimation of the number of miRNAs with target genes that were significantly up/downregulated or the number of target gene promoters that were significantly hypo/hyper methylated between any two brain regions

M $P^{\ell\ell'}_{mj,<}$s (or $P^{\ell\ell'}_{mj,>} = 1 - P^{\ell\ell'}_{mj,<}$ because we employed the Wilcoxon rank-sum test) were attributed to each of the miRNAs for one of the six pairwise comparisons of one sample and adjusted using the Benjaminhohenberg (BH) criterion. MiRNAs with adjusted P-values less than 0.05 were regarded as significant. This number was averaged over all the samples and the averaged value was taken as the estimation of the number of miRNAs with target genes that were significantly up/downregulated or the number of target gene promoters that were significantly hypo/hyper methylated between any two of the four brain regions.

1.4 Rank correlation coefficients between P-values attributed to miRNA-mediated regulation of target genes and P-values attributed to miRNA-targeting-specific promoter methylation

M $P^{\ell\ell'}_{mj,<}$s for one of the six pairwise comparisons of one sample was transformed to rank, $r^{\ell\ell'}_{mj}$. The rank, $r^{\ell\ell'}_{mj}$, attributed to each miRNA was averaged over all the samples,

$$
\begin{aligned}
r^{\ell\ell'}_{m} &\equiv \left\langle r^{\ell\ell'}_{mj} \right\rangle_j \\
\langle Q_j \rangle_j &\equiv \frac{1}{J} \sum_j Q_j
\end{aligned}
$$

where J is the total number of samples and Q_j is some variable attributed to the jth sample. The Spearman correlation coefficient of the averaged rank between the miRNA-mediated regulation of target genes and miRNA-targeting-specific promoter methylation is,

$$
\begin{aligned}
\rho^{mRNA,Methyl.}_{\ell\ell'} &\equiv \frac{\left\langle \Delta r^{\ell\ell',mRNA}_{0m} \cdot \Delta r^{\ell\ell',Methyl.}_{0m} \right\rangle_m}{\sqrt{\left\langle \left[\Delta r^{\ell\ell',mRNA}_{0m}\right]^2 \right\rangle_m} \sqrt{\left\langle \left[\Delta r^{\ell\ell',Methyl.}_{0m}\right]^2 \right\rangle_m}} \\
\Delta r^{\ell\ell',s}_{0m} &\equiv r^{\ell\ell',s}_{0m} - \langle r^{\ell\ell',s}_{0m} \rangle_m \\
\langle Q_m \rangle_m &\equiv \frac{1}{M} \sum_m Q_m,
\end{aligned}
$$

where M is number of miRNAs and Q_m is some variable attributed to the mth miRNA. $r^{\ell\ell',s}_{0m}$ is the rank order of $r^{\ell\ell',s}_{m}$ over $m = 1, \ldots, M$, where s is either mRNA or Methyl., representing mRNA expression and promoter methylation, respectively. P-values attributed to Spearman correlation coefficients were also computed.

Alternatively, the Spearman correlation coefficient of the averaged rank between the miRNA-mediated regulation of target genes and miRNA-targeting-specific promoter methylation was computed before aver-

aging,

$$\rho_{j\ell\ell'}^{mRNA,Methyl.} \equiv \frac{\left\langle \Delta r_{mj}^{\ell\ell',mRNA} \cdot \Delta r_{mj}^{\ell\ell',Methyl.} \right\rangle_m}{\sqrt{\left\langle \left[\Delta r_{mj}^{\ell\ell',mRNA}\right]^2 \right\rangle_m} \sqrt{\left\langle \left[\Delta r_{mj}^{\ell\ell',Methyl.}\right]^2 \right\rangle_m}}$$

$$\Delta r_{mj}^{\ell\ell',s} \equiv r_{mj}^{\ell\ell',s} - \langle r_{mj}^{\ell\ell',s} \rangle_m$$

Because the averaged correlation coefficient $\langle \rho_{j\ell\ell'}^{mRNA,Methyl.} \rangle_j$ was found to be almost zero (data not shown)—in other words positive and negative correlations appear with almost same probability—the standard deviation of the correlation coefficient was computed instead of the averaged value so as to express how many of the values that differed from zero were observed for the correlation coefficients,

$$\Delta \rho_{\ell\ell'}^{mRNA,Methyl.} \equiv \sqrt{\left\langle \left[\rho_{j\ell\ell'}^{mRNA,Methyl.} - \left\langle \rho_{j\ell\ell'}^{mRNA,Methyl.} \right\rangle_j \right]^2 \right\rangle_j}$$

1.5 Multiple regression model between miRNA-mediated regulation of target genes, miRNA-targeting-specific promoter methylation, and differential miRNA expression, with the additional consideration of age and gender

The proposed multiple regression model is

$$\log P_{mj,>}^{\ell\ell',mRNA} = A_m^{\ell\ell'} \cdot \log P_{mj,<}^{\ell\ell',Methyl.} + B_m^{\ell\ell'} \cdot \log \left(\frac{x_{mj\ell}}{x_{mj\ell'}} \right) + C_m^{\ell\ell'} \cdot \text{age}_j + D_m^{\ell\ell'} \cdot \text{gender}_j + E_m^{\ell\ell'}$$

where $P_{mj,>}^{\ell\ell',mRNA}$ and $P_{mj,<}^{\ell\ell',Methyl.}$ are the P-values attributed to miRNA-mediated regulation of target genes and miRNA-targeting-specific promoter methylation, respectively. $A_m^{\ell\ell'}, B_m^{\ell\ell'}, C_m^{\ell\ell'}, D_m^{\ell\ell'}$ and $E_m^{\ell\ell'}$ are all constants. $x_{mj\ell}$ is the mth miRNA expression of the ℓth region of the jth sample. age_j and gender_j are the age and gender of the jth sample respectively. gender_j =1 when the jth sample is from a male, otherwise gender_j=0. After the regression results were obtained, feature extraction based on Akaike's Information Criterion (AIC) was applied to choose the valid terms for the right hand side of this equation. This feature extraction procedure specifies the optimal combination of the terms with the minimum AIC values. A positive (negative) $A_m^{\ell\ell'}$ indicates a reciprocal (nonreciprocal) relationship between a miRNA and its target genes. Multiple regression and feature extraction were performed using the lm function in the R base package and the stepAIC function in the R MASS package [6], respectively.

1.6 Selection of miRNAs that significantly regulated target genes based on multiple regression models

The P-values that were computed based on the F test were attributed to each regression model of the miRNAs and adjusted using the BH criterion for each of the six pairwise comparisons of the four brain regions. Then the miRNAs with P-values that could be attributed to the constant $B_m^{\ell\ell'}$, the multiplier of $\log\left(\frac{x_{mj\ell}}{x_{mj\ell'}}\right)$, were selected if their P-values were less than 0.05 among the regression model with adjusted P-values that were less than 0.05.

1.7 KEGG pathway enrichment analysis of the selected miRNAs

The DIANA-mirpath (Ver. 2.0) web tool [7] was used to analyze the KEGG pathway enrichment of the miRNA target genes. Twelve groups of miRNAs (listed in Table 1), namely, the reciprocal and nonreciprocal miRNAs in each of the six pairwise comparisons, were uploaded to DIANA-mirpath one by one and P-values were obtained with the default parameter settings: union of target genes; target genes predicted by microT-CDS; P-value threshold as 0.05; microT threshold as 0.5; and FDR correction applied.

2 Supplementary Discussion

2.1 Supplementary Discussion on Selected KEGG pathways

2.1.1 TGF-β signaling pathway

Transforming growth factor (TGF)-β is known to play an important role in cell development and TGF-β signaling specifies the axons during brain development [8]. Thus, TGF-β signaling is important in brain development. However, TGF-β also plays several critical roles that are unrelated to development.

The TGF-β signaling pathway is known to be closely associated with brain disease and death. Bezchlibnyk *et al* [9] studied differential cDNA expression between post-mortem samples of frontal cortex tissue from patients with bipolar disorder and matched controls. They found that TGF-β1 decreased substantially in the bipolar disorder samples. Lippa *et al* [10] observed increased immunoreactivity of TGF-β type I (RI) and type II (RII) receptor subtypes in reactive glia in the frontal cortex and parahippocampal gyrus of Alzheimer's disease brains compared with matched controls. Plata-Salamán *et al* [11] showed that interleukin-1β (IL-1β), Tumor Necrosis Factor (TNF)-α and TGF-β1 mRNA levels were up-regulated in all brain regions following borna disease virus inoculation. Krieglstein *et al* [12] reported that glial cell line-derived neurotrophic factor failed to promote the survival of highly purified neuron populations *in vitro* unless it was supplemented with TGF-β.

Many reports have found a relationship between TGF-β and IL-1, and IL-1 is known to have diverse actions in the brain [13]. For example, da Cunha *et al* [14] showed that IL-1 stimulated the production

of TGF-β1 immunoreactive product in cells as well as TGF-β1 activity in culture fluids of all glial cells, astrocytes, microglial cells, and oligodendrocytes, derived from neonatal rat cortex and grown in cell type-enriched cultures. They also found that TGF-β1 production *in vitro* varied with the cell type and the isoform of IL-1. In addition, da Cunha *et al* [15] showed that amoeboid microglia, isolated and cultured from postmortem adult human brain, more frequently expressed TGF-β1 in the presence of IL-1. IL-1 is a cytokine that is elevated in the brain of Human Immunodeficiency Virus (HIV)-1-infected individuals compared with seronegative controls. Pasinetti *et al* [16] showed that TGF-β1 rapidly increased in adult rat brain in response to experimental lesions. Gayle *et al* [17] found that in both young and old rats, IL-1β induced a significant up-regulation of cerebellar IL-1 receptor antagonist (Ra), IL-1RI, and TGF-β1 mRNAs. These findings together indicate the importance of IL-1 in the brain, and imply that the TGF-β signaling pathway may also play a critical role in the brain.

Thus, it is likely that TGF-β plays critical roles in brain regions, and my finding that the miRNAs that target genes predicted to be involved in the TGF-β signaling pathway are differentially expressed between distinct brain regions, is a reasonable one.

2.1.2 Long-term potentiation and depression

Although the TGF-β pathway enrichment of the miRNA target genes is evidence that the miRNAs selected in this study can play critical roles in the brain, TGF-β is not a brain function-specific pathway.

Long-term potentiation and depression are more brain-specific. Long-term potentiation is a long-lasting enhancement in signal transmission between two neurons that results from stimulating them synchronously and long-term depression is an activity-dependent reduction in the efficacy of neuronal synapses lasting hours or longer following a long patterned stimulus. Prefrontal cortex long-term potentiation, but not long-term depression, has been found to be associated with the maintenance of extinction of learned fear in mice [18] and long-term depression is known to play important roles in cerebellar Purkinje cells [19].

The association of the long-term potentiation and long-term depression pathways with the miRNA target genes indicates that the selection of miRNAs was reasonable.

2.1.3 The mitogen-activated protein kinase (MAPK) signaling pathway

The MAPK pathway comprises a chain of proteins in the cell that communicates a signal from a receptor on the surface of the cell to the DNA in the nucleus of the cell. This pathway is known to play critical roles in brain regions. Yeste-Velasco *et al* showed that the MAPK pathway regulates neuronal apoptosis

through the phosphorylation of the retinoblastoma protein [20]. Bonni *et al* found that the MAPK-activated kinases were associated with Rsk-induced phosphorylation of the pro-apoptotic protein BAD at serine 112 which suppressed BAD-mediated apoptosis in neurons [21]. Wu *et al* reported an activity-dependent stabilization of the MAPK pathway that was prominent in hippocampal dendrites [22]. Schmid *et al* showed that MAPK activation was essential for the neural cell adhesion molecule L1-dependent neurite outgrowth [23]. Jover-Mengual *et al* showed that estradiol acted via the classical estrogen receptor, the insulin-like growth factor (IGF)-I receptor, and the ERK/MAPK signaling cascade to protect CA1 neurons against global brain ischemia in ovariectomized female rats and gerbils [24]. Li *et al* suggested that the synaptic activity of central nervous system neurons activated N-methyl-D-aspartate (NMDA) receptors that are the predominant molecular device for controlling synaptic plasticity and memory function, and which in turn stimulate translation from stored Wnt5a mRNA via the MAPK signaling pathway [25]. Wefers *et al* analyzed gene expression in the hippocampus and showed that nine downregulated MAPK target genes that were candidates to cause mutant mice to display the normal anxiety phenotype actually increased depression-like behavior [26].

The evidence clearly suggests that the MAPK signaling pathway plays a variety of critical roles in brain regions. The association of the MAPK pathway with the miRNA target genes again indicates that the selection of miRNAs in this study was reasonable.

2.1.4 mTOR signaling pathway

Recently, Magri and Galli reviewed the relationship between mTOR signaling pathway and neural stem cells [27] and Russo *et al* reviewed the cellular and molecular features of mTOR and related pathways and analyze their function in the brain [28]. Thus, the currently available evidence clearly shows that the mTOR signaling pathway is important in brain function and development.

2.1.5 Wnt signaling pathway

The Wnt signaling pathway is another network of proteins that passes signals from receptors on the surface of the cell to DNA in the nucleus. Although this pathway is thought to especially control cell-cell communication in the embryo and adult, numerous reports have described its importance in brain functions, particularly, those that are disease related.

For example, Shruster *et al* showed that the addition of Wnt3a increased the neuronal differentiation of amyloid $\beta(42)$-treated hippocampal progenitors, which inhibits the reduction of neural differentiation [29]. Munji *et al* found that the Wnt-β-catenin pathway (canonical Wnt pathway) regulated intermediate

progenitors differentiation into neurons [30]. Buchman reported that Wnt signaling was positively regulated by abnormal spindle microcephaly, mutations of which are known to be the most common cause of autosomal recessive primary microcephaly [31]. Rosen *et al* reported an adaptive role for altered Wnt signaling in progranulin deficiency-mediated frontotemporal dementia [32]. Peukert *et al* found that lack of Lhx2/Lhx9 function as well as increased Wnt signaling altered the expression of the thalamus-specific cell adhesion factor pcdh10b which subsequently led to a striking anterior-posterior disorganization of the caudal forebrain [33].

Although most of these studies are related to developmental diseases, they also show that a proper functioning Wnt signaling pathway is important for brain function. Thus, the enrichment of the Wnt signaling pathway among the miRNA target genes in the present study is consistent with previous studies.

2.1.6 ErbB signaling pathway

The ErbB signaling pathway is known to play critical roles during the various developmental stages and has also been shown to have a tight relationship with brain proliferation through the activation of neuregulin-1 (NRG-1) which is essential for the normal development of the nervous system.

Fallon *et al* found that constitutive activation of the NRG-1/erbB signaling pathway promoted the proliferation of SK-N-MC neuroepithelioma cells *in vitro* and hypothesized that NRG-1/erbB autocrine, paracrine or juxtacrine signaling may contribute to the development and/or progression of neuroepitheliomas *in vivo* [34]. Frohnert *et al* showed that NRG-1 isoforms and erbB kinases acted in an autocrine and/or paracrine fashion to promote mitogenesis in JS1 cells [35]. Stonecypher *et al* showed that neoplastic Schwann cells within neoplasms variably expressed the erbB kinases that mediating NRG-1 responses (erbB2, erbB3 and/or erbB4) [36, 37].

The reported mechanisms of the erbB signaling pathway, indicate that the erbB signaling pathway is rightly listed as one of the pathways associated with the miRNA target genes.

2.1.7 Lysine degradation

Lysine is known to play a critical role in memory formation and some reports have found that lysine has other functions as well.

Rao *et al* showed that lysine activity was high during the early stages of development [38]. Sauer *et al* demonstrated that a low L-lysine diet lowered the concentration of glutaric acid in the brain [39]. Pyridoxine dependent epilepsy has been shown to be caused by a defect of alpha-amino adipic semialdehyde dehydrogenase (antiquitin) in the cerebral lysine degradation pathway [40].

Although the number of studies is limited, it is evident that lysine may contribute to biological processes in brain other than memory formations.

2.1.8 N-glycan biosynthesis

Although N-glycan is not recognized as important for brain function, some reports have related its protein structure variants to brain function.

For example, N-glycan in the cerebrospinal fluid of eIF2B-mutated patient samples was found by Fogli *et al* to have an increased number of bi-antennary structures and a decreased number of tri-antennary/bisecting structures [41]. Ye and Marth studied N-glycan branching as a requirement in neuronal and postnatal viability [42]. Lee *et al* found that galectins bind the GlcNAc-branched N-glycans that are attached to glycoproteins on the surface of cells where they form a molecular lattice that restricts lateral movement and the endocytosis of glycoproteins [43].

Although these reports are related to protein structure and not to gene expression, N-glycan must be transcribed to be transformed. Thus, it is not strange that N-glycan structures differ between brain regions and the list in Table 2 includes miRNA that target the genes involved in N-glycan biosynthesis.

2.1.9 Tight junction and adherens junction

Götz and Huttner [44] have pointed out that tight junctions and adherens junctions are important structural components of the nervous system. Bauer *et al* found that neurons expressed the tight junctions-specific protein occludin, the main component of tight junctions [45]. Karaki *et al* reported that another component of tight junctions, claudin-4, was expressed by enteric neurons in the rat distal colon [46]. Devaux and Gow showed that claudin 11, which forms autotypic tight junctions that comprise the radial component of central nervous system myelin, affords rapid nerve conduction principally for small diameter myelinated axons [47]. Based upon their findings, Devaux and Gow also developed a computational model to more accurately represent conduction in small fibers [48]. Marthiens and Ffrench-Constant found that adherens junction domains were split by the asymmetric division of embryonic neural stem cells [49]. Meng and Takeuchi pointed out the importance of adherens junctions in the nervous system in their review of adherens junctions [50].

2.1.10 Regulation of the actin cytoskeleton

The actin cytoskeleton participates in many fundamental processes including the regulation of cell shape, motility, and adhesion [51]. It has also been reported that regulation of the actin cytoskeleton is important in brain. Sarmiere and Bamburg reviewed the actin cytoskeleton and discussed its modulation by the family of actin dynamizing proteins known as actin depolymerizing factor (ADF)/cofilin or AC proteins [52]. Regulation of actin dynamics by ADF/cofilin was also reported by Kuhn *et al* [53]. Dillon and Goda [54] and Cingolani and Goda [55] reviewed the dynamic actin cytoskeleton and emphasized its importance for morphological changes that are important for synapse functions .

2.1.11 Prion disease

Although diseases were not included explicitly in this study, all known prion diseases affect the structure of the brain or other neural tissue [56]. Indeed, it has been reported that neurons degenerate in prion disease [57]. Thus, the inclusion of miRNA target genes enriched in this pathway in Table 2 is not contradictory.

2.1.12 ECM-receptor interaction

The extracellular matrix (ECM) is known to play many critical roles in brain [58]. The ECM has been related to neurodegeneration [59] and has been reported to play especially important roles in the central nervous system [60].

2.1.13 Focal adhesion

Focal adhesions are sub-cellular macromolecular assemblies that mediate the regulatory effects (for example, cell anchorage) of ECM adhesion on cell behavior [61]. Some reports have emphasized the importance of focal adhesions in brain. Valiente *et al* showed that focal adhesion kinase (FAK) plays a fundamental role in the dynamic regulation of Gap-mediated adhesions during glial-guided neuronal migration in mouse [62]. Ferrari *et al* showed that focal adhesions and cell contractility stably link the topographical configuration of the extracellular environment to a corresponding neuronal polarity state [63]. Vallejo-Illarramendi *et al* demonstrated that FAK plays an essential role in cardiac outflow tract development by promoting the activation of molecules such as Crkl and Erk1/2 in neural crest cells [64]. Derkinderen *et al* suggested that a neuronal form of FAK was regulated by anandamide [65]. Chacón *et al* reported that FAK controlled filopodia formation and actin nucleation during axonal development [66].

2.1.14 Endocytosis

In neuronal cells, endocytosis is essential for recycling of the membrane after neurotransmitter release. Endocytosis also plays a critical role during the early developmental stages [67]. Kotowski *et al* found that endocytosis promoted rapid dopaminergic signaling in the central nervous system [68]. Raimondi *et al* reported an overlapping role of dynamin isoforms in synaptic vesicle endocytosis [69]. Tojima *et al* showed that asymmetric clathrin-mediated endocytosis drove repulsive growth cone guidance [70].

Although limited in number, these studies demonstrate the importance of endocytosis in brain functions.

2.1.15 Other pathways

Some of the other pathways listed in Table 2 may not be related directly to brain. For example, the several cancer/tumor-related pathways that include pancreatic cancer, pathways in cancer, melanoma, renal cell carcinoma, chronic myeloid leukemia, small cell lung cancer, non-small cell lung cancer, prostate cancer, thyroid cancer, and acute myeloid leukemia, were probably selected because of the tight relationship between miRNAs and cancers/tumors. Some other disease-related pathways such as type II diabetes mellitus, may also have been selected for similar reasons. Other pathways, for example, CAMS, cytokine–cytokine receptor interaction, and insulin signaling were recognized by Paul *et al* [71] as described above. More detailed experimental researches in the future should help validate the roles of other miRNAs in brain.

2.2 Divergence of the selected miRNAs and uniformity of the selected pathways

Most of the commonly selected KEGG pathways listed in Table 2 have some relationship with brain even though the selected miRNAs were not common to all four brain regions. This can be explained as follows: each miRNA can, theoretically, target hundreds of genes and each gene is targeted by several miRNAs and a specific combination of miRNAs can target the same set of genes.

For example, let us consider the TGF-β pathway for the cerebellum and frontal cortex pair. This pathway was enriched for the target genes of miRNAs whose target genes were significantly up/downregulated between the cerebellum and frontal cortex[a]. By definition, there were no overlaps between the miRNAs whose target genes are significantly upregulated and those whose target genes are significantly downregulated. Despite this, in the TGF-β pathway, some of the target genes of miRNAs whose target genes are significantly upregulated are the same as the target genes of miRNAs whose target genes are significantly downregulated. A total of 31 differentially regulated genes corresponding to 36 mRNAs with refseq IDs were selected[b]. Of the 36 mRNAs, 25 mRNAs were significantly up/downregulated between cerebellum and frontal cortex (Table

3). For example, SMAD5 and RBL2 were significantly more expressed in cerebellum than frontal cortex ($P = 5.54 \times 10^{-23}$ and $P = 1.22 \times 10^{-22}$), respectively. SMAD5 is known to be activated during brain development [72] and RBL2, also known as RB2, is known to function in neural differentiation [73]. This is only one example, but it illustrates why different combinations of miRNAs can target genes in the same KEGG pathway.

The TGF-β pathway was not selected every time a substantial number of miRNAs were considered. For example, for a set of five miRNAs for which the target genes were downregulated between pons and temporal cortex, the only pathway that was found was for valine, leucine and isoleucine biosynthesis. Conversely, for a set of seven miRNAs for which the target genes were downregulated between cerebellum and frontal cortex, 29 pathways were selected including the TGF-β signaling pathway. This finding shows that the TGF-β signaling pathway was not selected automatically just because the number of selected miRNAs was substantial. Thus, in the context of this analysis we can understand why commonly selected pathways were found despite the fact that selected miRNAs were not common.

2.3 Positive and negative correlations between miRNA expression and target gene expression

Although some of the miRNA-mediated regulation of target genes was not reciprocal, this apparent contradiction was not always caused by a failure in the analysis because, even in miRNA transfection experiments, some miRNA target genes were found to be upregulated after miRNA transfection [74].

2.4 Possible biological reasons for cooperative regulation of target genes by miRNAs and promoter methylation

Cooperative regulation of mRNAs by miRNA and promoter methylation has not been widely investigated, partly because no direct relationship between miRNA function and promoter methylation seemed to exist. The miRNA-mediated regulation of target genes takes place in the cytoplasm, while promoter methylation occurs in the nucleus of cells. These two processes, therefore, are temporally and spatially apart. However, the relationship between TFs and miRNAs has been a popular topic of study and TFs work in the nucleus while miRNAs work in the cytoplasm. A major difference between TFs and methylation is the few number of known proteins that mediate promoter methylation as against the many known TF families. If promoter methylation cooperatively regulates gene expression with miRNAs of which there is a large number of species, it is interesting to speculate about the mechanisms that may be involved.

Halytskiy [75] suggested that Ago protein binding to miRNA may take place in the nucleus and induce

promoter methylation. Recently, it has been suggested that Ago protein can function in the nucleus [76] and Weinmann *et al* has reported that Ago protein was localized in the nucleus [77]. If Ago protein binding miRNA can directly methylate promoters in the nucleus, then this mechanism may be able to mediate miRNA-targeting-specific promoter methylation. Further studies into this possibility are required to confirm these findings.

References

1. Yoshizawa M, Taguchi YH, Yasuda J: **Inference of gene regulation via miRNAs during ES cell differentiation using MiRaGE method**. *Int J Mol Sci* 2011, **12**(12):9265–9276.
2. Taguchi YH: **Inference of Target Gene Regulation via miRNAs during Cell Senescence by Using the MiRaGE Server**. *Aging Dis* 2012, **3**(4):301–306.
3. Taguchi Yh: **Competitive target gene regulation by promoter methylation and miRNA**. *IPSJ SIG Technical Reports* 2012, **2012**:1–6, [http://ci.nii.ac.jp/naid/110009459611/en/].
4. Taguchi Yh: **Inference of the target gene regulation by miRNA via MiRaGE Server**. In *Introduction to Sequence and Genome Analysis*. Edited by iConcept Press Ltd, Hong Kong: iConcept Press 2012:in press.
5. **MiRaGE** [http://www.bioconductor.org/packages/release/bioc/html/MiRaGE.html].
6. R Core Team: *R: A Language and Environment for Statistical Computing*. R Foundation for Statistical Computing, Vienna, Austria 2012, [http://www.R-project.org/]. [ISBN 3-900051-07-0].
7. Vlachos IS, Kostoulas N, Vergoulis T, Georgakilas G, Reczko M, Maragkakis M, Paraskevopoulou MD, Prionidis K, Dalamagas T, Hatzigeorgiou AG: **DIANA miRPath v.2.0: investigating the combinatorial effect of microRNAs in pathways**. *Nucleic Acids Res.* 2012, **40**(Web Server issue):498–504.
8. Yi JJ, Barnes AP, Hand R, Polleux F, Ehlers MD: **TGF-beta signaling specifies axons during brain development**. *Cell* 2010, **142**:144–157.
9. Bezchlibnyk YB, Wang JF, McQueen GM, Young LT: **Gene expression differences in bipolar disorder revealed by cDNA array analysis of post-mortem frontal cortex**. *J. Neurochem.* 2001, **79**(4):826–834.
10. Lippa CF, Flanders KC, Kim ES, Croul S: **TGF-β receptors-I and -II immunoexpression in Alzheimer's disease: a comparison with aging and progressive supranuclear palsy**. *Neurobiol. Aging* 1998, **19**(6):527–533.
11. Plata-Salamán CR, Ilyin SE, Gayle D, Romanovitch A, Carbone KM: **Persistent Borna disease virus infection of neonatal rats causes brain regional changes of mRNAs for cytokines, cytokine receptor components and neuropeptides**. *Brain Res. Bull.* 1999, **49**(6):441–451.
12. Krieglstein K, Strelau J, Schober A, Sullivan A, Unsicker K: **TGF-beta and the regulation of neuron survival and death**. *J. Physiol. Paris* 2002, **96**(1-2):25–30.
13. Rothwell NJ, Luheshi GN: **Interleukin 1 in the brain: biology, pathology and therapeutic target**. *Trends Neurosci.* 2000, **23**(12):618–625.
14. da Cunha A, Jefferson JA, Jackson RW, Vitković L: **Glial cell-specific mechanisms of TGF-β1 induction by IL-1 in cerebral cortex**. *J. Neuroimmunol.* 1993, **42**:71–85.
15. da Cunha A, Jefferson JJ, Tyor WR, Glass JD, Jannotta FS, Cottrell JR, Resau JH: **Transforming growth factor-β1 in adult human microglia and its stimulated production by interleukin-1**. *J. Interferon Cytokine Res.* 1997, **17**(11):655–664.
16. Pasinetti GM, Nichols NR, Tocco G, Morgan T, Laping N, Finch CE: **Transforming growth factor β1 and fibronectin messenger RNA in rat brain: responses to injury and cell-type localization**. *Neuroscience* 1993, **54**(4):893–907.
17. Gayle D, Ilyin SE, Romanovitch AE, Peloso E, Satinoff E, Plata-Salaman CR: **Basal and IL-1beta-stimulated cytokine and neuropeptide mRNA expression in brain regions of young and old Long-Evans rats**. *Brain Res. Mol. Brain Res.* 1999, **70**:92–100.
18. Herry C, Garcia R: **Prefrontal cortex long-term potentiation, but not long-term depression, is associated with the maintenance of extinction of learned fear in mice**. *J. Neurosci.* 2002, **22**(2):577–583.
19. Ito M: **Cerebellar long-term depression: characterization, signal transduction, and functional roles**. *Physiol. Rev.* 2001, **81**(3):1143–1195.
20. Yeste-Velasco M, Folch J, Pallas M, Camins A: **The p38(MAPK) signaling pathway regulates neuronal apoptosis through the phosphorylation of the retinoblastoma protein**. *Neurochem. Int.* 2009, **54**(2):99–105.
21. Bonni A, Brunet A, West AE, Datta SR, Takasu MA, Greenberg ME: **Cell survival promoted by the Ras-MAPK signaling pathway by transcription-dependent and -independent mechanisms**. *Science* 1999, **286**(5443):1358–1362.

22. Wu GY, Deisseroth K, Tsien RW: **Spaced stimuli stabilize MAPK pathway activation and its effects on dendritic morphology**. *Nat. Neurosci.* 2001, **4**(2):151–158.

23. Schmid RS, Pruitt WM, Maness PF: **A MAP kinase-signaling pathway mediates neurite outgrowth on L1 and requires Src-dependent endocytosis**. *J. Neurosci.* 2000, **20**(11):4177–4188.

24. Jover-Mengual T, Zukin RS, Etgen AM: **MAPK signaling is critical to estradiol protection of CA1 neurons in global ischemia**. *Endocrinology* 2007, **148**(3):1131–1143.

25. Li Y, Li B, Wan X, Zhang W, Zhong L, Tang SJ: **NMDA receptor activation stimulates transcription-independent rapid wnt5a protein synthesis via the MAPK signaling pathway**. *Mol Brain* 2012, **5**:1.

26. Wefers B, Hitz C, Holter SM, Trumbach D, Hansen J, Weber P, Putz B, Deussing JM, de Angelis MH, Roenneberg T, Zheng F, Alzheimer C, Silva A, Wurst W, Kuhn R: **MAPK signaling determines anxiety in the juvenile mouse brain but depression-like behavior in adults**. *PLoS ONE* 2012, **7**(4):e35035.

27. Magri L, Galli R: **mTOR signaling in neural stem cells: from basic biology to disease**. *Cel Mol Life Sci* 2012, :1–12, [http://dx.doi.org/10.1007/s00018-012-1196-x].

28. Russo E, Citraro R, Constanti A, De Sarro G: **The mTOR Signaling Pathway in the Brain: Focus on Epilepsy and Epileptogenesis**. *Mol. Neurobiol.* 2012, **46**(3):662–681.

29. Shruster A, Eldar-Finkelman H, Melamed E, Offen D: **Wnt signaling pathway overcomes the disruption of neuronal differentiation of neural progenitor cells induced by oligomeric amyloid β-peptide**. *J. Neurochem.* 2011, **116**(4):522–529.

30. Munji RN, Choe Y, Li G, Siegenthaler JA, Pleasure SJ: **Wnt signaling regulates neuronal differentiation of cortical intermediate progenitors**. *J. Neurosci.* 2011, **31**(5):1676–1687.

31. Buchman JJ, Durak O, Tsai LH: **ASPM regulates Wnt signaling pathway activity in the developing brain**. *Genes Dev.* 2011, **25**(18):1909–1914.

32. Rosen EY, Wexler EM, Versano R, Coppola G, Gao F, Winden KD, Oldham MC, Martens LH, Zhou P, Farese RV, Geschwind DH: **Functional genomic analyses identify pathways dysregulated by progranulin deficiency, implicating Wnt signaling**. *Neuron* 2011, **71**(6):1030–1042.

33. Peukert D, Weber S, Lumsden A, Scholpp S: **Lhx2 and Lhx9 determine neuronal differentiation and compartition in the caudal forebrain by regulating Wnt signaling**. *PLoS Biol.* 2011, **9**(12):e1001218.

34. Fallon KB, Havlioglu N, Hamilton LH, Cheng TP, Carroll SL: **Constitutive activation of the neuregulin-1/erbB signaling pathway promotes the proliferation of a human peripheral neuroepithelioma cell line**. *J. Neurooncol.* 2004, **66**(3):273–284.

35. Frohnert PW, Stonecypher MS, Carroll SL: **Constitutive activation of the neuregulin-1/ErbB receptor signaling pathway is essential for the proliferation of a neoplastic Schwann cell line**. *Glia* 2003, **43**(2):104–118.

36. Stonecypher MS, Byer SJ, Grizzle WE, Carroll SL: **Activation of the neuregulin-1/ErbB signaling pathway promotes the proliferation of neoplastic Schwann cells in human malignant peripheral nerve sheath tumors**. *Oncogene* 2005, **24**(36):5589–5605.

37. Stonecypher MS, Chaudhury AR, Byer SJ, Carroll SL: **Neuregulin growth factors and their ErbB receptors form a potential signaling network for schwannoma tumorigenesis**. *J. Neuropathol. Exp. Neurol.* 2006, **65**(2):162–175.

38. Rao VV, Pan X, Chang YF: **Developmental changes of L-lysine-ketoglutarate reductase in rat brain and liver**. *Comp. Biochem. Physiol., B* 1992, **103**:221–224.

39. Sauer SW, Opp S, Hoffmann GF, Koeller DM, Okun JG, Kolker S: **Therapeutic modulation of cerebral L-lysine metabolism in a mouse model for glutaric aciduria type I**. *Brain* 2011, **134**(Pt 1):157–170.

40. Mills PB, Struys E, Jakobs C, Plecko B, Baxter P, Baumgartner M, Willemsen MA, Omran H, Tacke U, Uhlenberg B, Weschke B, Clayton PT: **Mutations in antiquitin in individuals with pyridoxine-dependent seizures**. *Nat. Med.* 2006, **12**(3):307–309.

41. Fogli A, Merle C, Roussel V, Schiffmann R, Ughetto S, Theisen M, Boespflug-Tanguy O: **CSF N-glycan profiles to investigate biomarkers in brain developmental disorders: application to leukodystrophies related to eIF2B mutations**. *PLoS ONE* 2012, **7**(8):e42688.

42. Ye Z, Marth JD: **N-glycan branching requirement in neuronal and postnatal viability**. *Glycobiology* 2004, **14**(6):547–558.

43. Lee SU, Grigorian A, Pawling J, Chen IJ, Gao G, Mozaffar T, McKerlie C, Demetriou M: **N-glycan processing deficiency promotes spontaneous inflammatory demyelination and neurodegeneration**. *J. Biol. Chem.* 2007, **282**(46):33725–33734.

44. Gotz M, Huttner WB: **The cell biology of neurogenesis**. *Nat. Rev. Mol. Cell Biol.* 2005, **6**(10):777–788.

45. Bauer H, Stelzhammer W, Fuchs R, Weiger TM, Danninger C, Probst G, Krizbai IA: **Astrocytes and neurons express the tight junction-specific protein occludin in vitro**. *Exp. Cell Res.* 1999, **250**(2):434–438.

46. Karaki S, Kaji I, Otomo Y, Tazoe H, Kuwahara A: **The tight junction component protein, claudin-4, is expressed by enteric neurons in the rat distal colon**. *Neurosci. Lett.* 2007, **428**(2-3):88–92.

47. Devaux J, Gow A: **Tight junctions potentiate the insulative properties of small CNS myelinated axons**. *J. Cell Biol.* 2008, **183**(5):909–921.

48. Gow A, Devaux J: **A model of tight junction function in central nervous system myelinated axons**. *Neuron Glia Biol.* 2008, **4**(4):307–317.

49. Marthiens V, ffrench Constant C: **Adherens junction domains are split by asymmetric division of embryonic neural stem cells**. *EMBO Rep.* 2009, **10**(5):515–520.

50. Meng W, Takeichi M: **Adherens junction: molecular architecture and regulation**. *Cold Spring Harb Perspect Biol* 2009, **1**(6):a002899.

51. Roffers-Agarwal J, Xanthos JB, Miller JR: **Regulation of actin cytoskeleton architecture by Eps8 and Abi1**. *BMC Cell Biol.* 2005, **6**:36.

52. Sarmiere PD, Bamburg JR: **Regulation of the neuronal actin cytoskeleton by ADF/cofilin**. *J. Neurobiol.* 2004, **58**:103–117.

53. Kuhn TB, Meberg PJ, Brown MD, Bernstein BW, Minamide LS, Jensen JR, Okada K, Soda EA, Bamburg JR: **Regulating actin dynamics in neuronal growth cones by ADF/cofilin and rho family GTPases**. *J. Neurobiol.* 2000, **44**(2):126–144.

54. Dillon C, Goda Y: **The actin cytoskeleton: integrating form and function at the synapse**. *Annu. Rev. Neurosci.* 2005, **28**:25–55.

55. Cingolani LA, Goda Y: **Actin in action: the interplay between the actin cytoskeleton and synaptic efficacy**. *Nat. Rev. Neurosci.* 2008, **9**(5):344–356.

56. Budka H: **Neuropathology of prion diseases**. *Br. Med. Bull.* 2003, **66**:121–130.

57. Liberski PP, Gajdusek DC, Brown P: **How do neurons degenerate in prion diseases or transmissible spongiform encephalopathies (TSEs): neuronal autophagy revisited**. *Acta Neurobiol Exp (Wars)* 2002, **62**(3):141–147.

58. Ruoslahti E: **Brain extracellular matrix**. *Glycobiology* 1996, **6**(5):489–492.

59. Bonneh-Barkay D, Wiley CA: **Brain extracellular matrix in neurodegeneration**. *Brain Pathol.* 2009, **19**(4):573–585.

60. Zimmermann DR, Dours-Zimmermann MT: **Extracellular matrix of the central nervous system: from neglect to challenge**. *Histochem. Cell Biol.* 2008, **130**(4):635–653.

61. Chen CS, Alonso JL, Ostuni E, Whitesides GM, Ingber DE: **Cell shape provides global control of focal adhesion assembly**. *Biochem. Biophys. Res. Commun.* 2003, **307**(2):355–361.

62. Valiente M, Ciceri G, Rico B, Marin O: **Focal adhesion kinase modulates radial glia-dependent neuronal migration through connexin-26**. *J. Neurosci.* 2011, **31**(32):11678–11691.

63. Ferrari A, Cecchini M, Serresi M, Faraci P, Pisignano D, Beltram F: **Neuronal polarity selection by topography-induced focal adhesion control**. *Biomaterials* 2010, **31**(17):4682–4694.

64. Vallejo-Illarramendi A, Zang K, Reichardt LF: **Focal adhesion kinase is required for neural crest cell morphogenesis during mouse cardiovascular development**. *J. Clin. Invest.* 2009, **119**(8):2218–2230.

65. Derkinderen P, Toutant M, Burgaya F, Le Bert M, Siciliano JC, de Franciscis V, Gelman M, Girault JA: **Regulation of a neuronal form of focal adhesion kinase by anandamide**. *Science* 1996, **273**(5282):1719–1722.

66. Chacon MR, Navarro AI, Cuesto G, del Pino I, Scott R, Morales M, Rico B: **Focal adhesion kinase regulates actin nucleation and neuronal filopodia formation during axonal growth**. *Development* 2012, **139**(17):3200–3210.

67. Parton RG, Dotti CG: **Cell biology of neuronal endocytosis**. *J. Neurosci. Res.* 1993, **36**:1–9.

68. Kotowski SJ, Hopf FW, Seif T, Bonci A, von Zastrow M: **Endocytosis promotes rapid dopaminergic signaling**. *Neuron* 2011, **71**(2):278–290.

69. Raimondi A, Ferguson SM, Lou X, Armbruster M, Paradise S, Giovedi S, Messa M, Kono N, Takasaki J, Cappello V, O'Toole E, Ryan TA, De Camilli P: **Overlapping role of dynamin isoforms in synaptic vesicle endocytosis**. *Neuron* 2011, **70**(6):1100–1114.

70. Tojima T, Itofusa R, Kamiguchi H: **Asymmetric clathrin-mediated endocytosis drives repulsive growth cone guidance**. *Neuron* 2010, **66**(3):370–377.

71. Paul A, Cai Y, Atwal GS, Huang ZJ: **Developmental coordination of gene expression between synaptic partners during GABAergic circuit assembly in cerebellar cortex**. *Frontiers in Neural Circuits* 2012, **6**(37), [http://www.frontiersin.org/neural_circuits/10.3389/fncir.2012.00037/abstract].

72. Lopez-Coviella I, Mellott TM, Kovacheva VP, Berse B, Slack BE, Zemelko V, Schnitzler A, Blusztajn JK: **Developmental pattern of expression of BMP receptors and Smads and activation of Smad1 and Smad5 by BMP9 in mouse basal forebrain**. *Brain Res.* 2006, **1088**:49–56.

73. Jori FP, Melone MA, Napolitano MA, Cipollaro M, Cascino A, Giordano A, Galderisi U: **RB and RB2/p130 genes demonstrate both specific and overlapping functions during the early steps of in vitro neural differentiation of marrow stromal stem cells**. *Cell Death Differ.* 2005, **12**:65–77.

74. Shahab S, Matyunina L, Hill C, Wang L, Mezencev R, Walker L, McDonald J: **The effects of MicroRNA transfections on global patterns of gene expression in ovarian cancer cells are functionally coordinated**. *BMC Medical Genomics* 2012, **5**:33, [http://www.biomedcentral.com/1755-8794/5/33].

75. Halytskiy V: **Hypothesis of initiation of DNA methylation de novo and allelic exclusion by small RNAs**. *Cell and Tissue Biology* 2008, **2**:97–106, [http://dx.doi.org/10.1134/S1990519X08020016].

76. Ender C, Meister G: **Argonaute proteins at a glance**. *J. Cell. Sci.* 2010, **123**(Pt 11):1819–1823.

77. Weinmann L, Hock J, Ivacevic T, Ohrt T, Mutze J, Schwille P, Kremmer E, Benes V, Urlaub H, Meister G: **Importin 8 is a gene silencing factor that targets argonaute proteins to distinct mRNAs**. *Cell* 2009, **136**(3):496–507.

2.5 Table 3 - Genes differently expressed between frontal cortex and cerebellum in TFG-β pathway

Genes included in TGF-β pathway whose mean expression in cerebellum (CRBLM) is significantly greater (a) or smaller (b) than that in frontal cortex (FCTX). Selected genes are common for both reciprocal and nonreciprocal relathionship between miRNA and target genes. The values in columns labelled as CRBLM and FCTX indicate mean expression over samples, respectively. p-values were computed based upon two-sided t test and q-values were adjusted based on BH criterion. More than one refseq IDs were sometimes attributed to one gene symbol so as to represents distinct isoforms.

(a) CRBLM > FCTX

Gene symbol	Refseq mRNA	CBRLM	FCTX	p-value	q-value
ZFYVE16	NM_014733	379.10	315.78	3.12e-05	8.24e-05
ACVR2A	NM_001616	301.67	249.68	3.03e-06	9.36e-06
SMAD5	NM_001001420	2070.75	1050.82	5.54e-23	6.83e-22
ACVR2B	NM_001106	263.49	221.02	2.74e-03	5.07e-03
TGFBR1	NM_004612	179.61	166.86	1.57e-03	3.24e-03
SP1	NM_138473	644.87	321.13	2.60e-30	4.81e-29
E2F4	NM_001950	895.05	737.76	6.78e-10	2.51e-09
RBL2	NM_005611	1325.18	813.88	1.22e-22	1.13e-21
RPS6KB1	NM_003161	1874.87	1453.88	6.54e-07	2.20e-06
ROCK1	NM_005406	229.47	189.64	3.85e-14	2.03e-13

(b) CRBLM < FCTX

Gene symbol	Refseq mRNA	CBRLM	FCTX	p-value	q-value
BMPR2	NM_001204	755.95	1081.43	1.14e-10	4.68e-10
TGFB2	NM_003238	146.80	173.75	9.98e-13	4.62e-12
PITX2	NM_000325	133.85	142.76	8.20e-03	1.32e-02
PPP2CA	NM_002715	6185.02	7170.70	1.52e-04	3.52e-04
SMAD5	NM_001001419	133.94	141.30	2.66e-02	3.93e-02
ID2	NM_002166	1224.03	2262.97	1.62e-21	1.18e-20
TGFBR2	NM_003242	419.08	509.84	2.03e-02	3.13e-02
TGFBR2	NM_001024847	138.58	148.31	4.63e-05	1.14e-04
ID3	NM_002167	507.03	732.96	1.17e-03	2.56e-03
MAPK1	NM_002745	794.07	1253.35	1.91e-21	1.18e-20
MAPK1	NM_138957	214.98	234.92	1.15e-05	3.26e-05
SMAD2	NM_005901	127.55	136.03	5.12e-03	8.61e-03
LTBP1	NM_206943	140.04	150.27	2.50e-03	4.87e-03
SMAD3	NM_005902	411.53	947.52	6.84e-39	2.53e-37
CUL1	NM_003592	1157.79	1235.39	3.28e-03	5.78e-03
